# DNA methylation as an early diagnostic marker of cancer (Review)

**DOI:** 10.3892/br.2014.237

**Published:** 2014-02-14

**Authors:** YUANYUAN DONG, HAIYANG ZHAO, HAIYAN LI, XIAOKUN LI, SHULIN YANG

**Affiliations:** 1 School of Environmental and Biological Engineering, Nanjing University of Science and Technology, Nanjing, Jiangsu 210094, P.R. China; 2 Engineering Research Center of Bioreactor and Pharmaceutical Development, Ministry of Education, Jilin Agricultural University, Changchun, Jilin 130118, P.R. China

**Keywords:** DNA methylation, molecular marker, early diagnosis

## Abstract

DNA methylation is one of the essential epigenetic mechanisms that are closely correlated with the mechanisms underlying cell growth, differentiation and transformation in eukaryotes. Global changes in the epigenetic landscape are considered to be a hallmark of cancer. The initiation and progression of cancer are mediated through epigenetic modifications along with genetic alterations. Aberrant methylation of promoter regions is an epigenetic abnormality of the human genome that is highly characteristic of cancer. In this review, we aimed to summarize our current understanding of the alterations in the epigenetic landscape and investigate the potential use of DNA and RNA methylation in effective molecular treatment strategies.

## 1. Introduction

Complex diseases, such as malignant tumors and diabetes, are a common occurrence and represent a major public health concern. Despite the significant advances in cancer treatment, the overall cancer-related mortality is ~90%, due to late-stage diagnosis and failure to optimize treatment. Therefore, effective biomarkers for cancer diagnosis are urgently needed.

Cancers such as lung, colon and breast cancer are frequently diagnosed at a late stage. Despite intensified efforts focused on improving the survival of cancer patients, only a moderate improvement is generally achieved. Failure of early diagnosis often leads to low treatment efficiency and poor prognosis; thus, the identification of a signal characterizing the early stages of formation and progression of cancer may reduce the incidence of this disease (1). Early findings of clinical studies indicated that early detection may offer a variety of novel efficient and cost-effective opportunities for cancer treatment (2).

The mechanisms underlying cancer development are complicated and cancer was originally perceived as a genetic disease. However, it was previously demonstrated that the initiation and progression of cancer involve epigenetic abnormalities, such as DNA methylation, histone modifications, nucleosome positioning and aberrant expression of non-coding RNAs, specifically microRNAs (3–7). The term ‘epigenetics’ was originally used by Waddington in 1939 to describe the causal interactions between genes and phenotype, including alterations in chromatin structure and their effects on gene function. DNA methylation is currently deemed to be one of the essential epigenetic mechanisms (8). Failure of proper maintenance of epigenetic marks may result in inappropriate activation or inhibition of various signaling pathways, leading to disease states, such as cancer. Aberrant DNA hypermethylation often occurs in the promoter regions of specific transcription factors that are involved in the formation and progression of malignant tumors. Accordingly, DNA methylation may be a biomarker of early cancer detection. In this study, we briefly reviewed the correlation between DNA methylation and early disease diagnosis.

## 2. Epigenetics and DNA methylation

Epigenomes comprise all genome-wide chromatin modifications, including covalent modification of DNA via methylation, histone modifications and regulation of small RNAs. Unlike DNA alterations that may constitute an inheritable mechanism, epigenetics represent a labile and dynamic mechanism for the regulation of cell-specific gene expression patterns, adaptation of response to environmental factors and mediation of disease development. It was demonstrated that epigenomic variability leads to susceptibility to disease, even among individuals with identical genetic information (9). Epigenomes are potentially significant for the comprehensive understanding of cell development, tissue differentiation and disease biogenesis. In general, the process of epigenetics mainly refers to chromatin structure alterations that comprise DNA methylation, histone modifications and RNA interference. DNA methylation and histone modifications may modulate the interaction of *cis*-acting elements with *trans*-acting factors and gene expression. Various epigenetic experiments, involving genomic imprinting (10), manner of monoallelic expression (11) and high-throughput sequencing technology (12), are currently available and commonly used to gain insight into this phenomenon.

DNA methylation is a common epigenetic modification involving the methylation of 5′-cytosine residues by methyltransferases and is often detected in the dinucleotides of CpG sequences. Approximately 70–80% of the CpG dinucleotides undergo methylation and are referred to as ‘CpG islands’, which are abundant in the human genome (13). Methylation is often localized in promoter regions and occasionally in transcriptional regulatory regions in mammals, plants and even prokaryotes (14,15). A number of studies indicated that DNA methylation is crucial in the regulation of transcriptional silencing and transcription potential (16,17). Aberrant methylation of promoters in eukaryotic cells may lead to silencing of important genes, such as tumor suppressor genes, affecting their related transcriptional pathways and ultimately leading to the development of disease.

## 3. Process of DNA methylation

DNA methylation may be classified as hyper- and hypomethylation, according to increased and decreased levels of genomic modification, respectively. Hypermethylation is an epigenetic alteration often leading to gene-inactivating deletions and translocations. Hypermethylated cells may exhibit a phenotype of drug-resistance (18) or malignant proliferation. Wu *et al* (19) demonstrated that 16 genes, including *BMP4*, *POU4F3* and *GDNF*, are frequently hypermethylated in tumors. The other alteration is hypomethylation (reduced level or loss of methylation compared to that in normal cells). Chromosome remodeling or reactivation of transposable elements was generated via demethylation of exons and introns or repetitive DNA sequences. An increased number of DNA hypomethylation loci in CpGs was shown to increase chromosomal instability and oncogene activation. For example, the hypomethylation of LINE-1 and Alu retrotransposons is frequently associated with lung cancer (20), the hypomethylation of the mesothelin promoter may contribute to the development of malignant mesothelioma (21) and DNA hypermethylation was previously described in peritoneal mesothelioma (22). DNA hypomethylation *per se* may induce complex disease. However, a single specific mechanism associating DNA hypomethylation with cancer has not yet been identified.

## 4. Detection methods of disease-associated DNA methylation

Complicated diseases have been associated with the methylation status. Previous studies on cardiovascular and neurodegenerative diseases, by Stenvinkel *et al* (23) and Obeid *et al* (24), respectively, reported that DNA methylation is involved in major human pathologies. Investigating the potential and practical methods of DNA methylation is essential for determining whether there is an association between aberrant DNA methylation within CpG-rich sequences and cancer.

Several different methods have been proposed for DNA methylation analysis and some of the advantages and disadvantages of the different approaches, particularly concentrating on genome-scale DNA methylation, were outlined based on enzymes combined with PCR methods. Conducting a DNA methylation analysis requires a highly precise and accurate determination of the methylation status. Several methods based on PCR have been developed to evaluate the methylation level of genes. Bisulphite treatment and PCR amplification are used for locus-specific detection. For example, a quantitative methylation-specific PCR assay was developed for high-throughput analysis and a real-time assay for individual methylated targets. Assays of other useful techniques may be applied to genes with 5-methylcytosine (m5C). The distribution of m5C within DNA is unique and may be used for genome-scale methylation analysis (25). For example, restriction landmark genome scanning was the first DNA methylation profiling technique that was widely used in identifying methylated loci in species or in a tissue-specific manner. Chromatin immunoprecipitation, based on microarray or next-generation sequencing, used antibodies or methyl-binding proteins for massive methylated DNA profiling. These powerful approaches provide accurate, reproducible and sensitive data in comprehensive methylation epigenomic and genomic typing.

## 5. DNA methylation as a biomarker for early cancer diagnosis

As a specific pattern of gene expression in mammals, DNA methylation is essential for tissue development. Abnormal DNA methylation commonly disrupts molecular signaling mechanisms and leads to the development of various diseases, such as cancer. DNA methylation was the first epigenetic alteration to be identified in cancer (26). Therefore, it is considered to be a hallmark of cancer, it is detected in several types of cancer cells, including colon, breast, ovarian and cervical cancer cells and is associated with alterations in specific gene expression.

### DNA methylation and cancer

The DNA methylation status is correlated with cancer and the methylation level is inversely correlated with mRNA expression levels. Jin *et al* (27) reported that the risk of Barrett’s esophagus (BE) progressing into esophageal adenocarcinoma is 30- to 125-fold higher compared to the general population. Methylation assays were performed in 195 subjects with BE to evaluate the methylation levels and frequencies of individual genes, including *p16*, *RUNX3*, *HPP1*, *NELL1*, *TAC1*, *SST*, *AKAP12* and *CDH13* and five of these genes (*NELL1*, *TAC1*, *SST*, *AKAP12* and *CDH13*) were found to harbour methylated sites (27). In breast cancer patients, the patterns of methylation of the *ESR1* and *14-3-3-σ* promoters were significantly different compared to healthy controls (28). Glockner *et al* (29) reported that methylation of the tissue factor pathway inhibitor 2 gene was frequently detected in 171 samples from human colorectal cancers. Therefore, the aberrant methylation or hypermethylation of these promoters led to the dysregulated expression of cancer-related genes, facilitating the development of malignant tumors. In addition to the methylation of cancer-related genes, the genome-wide m5C levels of leukocyte DNA are also independently associated with breast cancer. Choi *et al* (30) compared 176 breast cancer cases with 173 healthy controls and demonstrated that the m5C levels were significantly lower in breast cancer.

### DNA methylation associated with cancer diagnosis

The identification of patients with organ-confined carcinoma is key to early-stage diagnosis of cancer. Common organ-confined cancers, such as lung, hepatic, breast, cervical, colorectal and genitourinary tract cancers, result in patient death due to the lack of effective clinical diagnosis. In these cancers, the methylation of the promoter regions of tumor suppressor genes, such as *CDH1*, *APC*, *MGMT*, *RASSF1A*, *GSTP1*, *p16* and *RAR-β2*, affect the activity of tumor suppressor genes, typically leading to transcriptional silencing. A number of important genes that undergo silencing interfere with important cancer-related cell pathways. Thus, the aberrant methylation of the promoters of cancer-related genes may be deemed as a potential biomarker contributing to early cancer detection and prediction of prognosis.

A series of novel DNA methylation biomarkers in the plasma and stool were developed for various detection purposes. Vimentin is transcriptionally silent in normal epithelia and aberrant vimentin expression has been used as a cancer marker in fecal DNA testing (31). In 2005, vimentin was licensed by the US Food and Drug Administration (FDA) for colorectal cancer (CRC) diagnosis. Another CRC-related gene, *SEPT9*, is commonly detected in the plasma of patients with primary CRC and was submitted to the FDA for marketing application in 2010 as a molecular marker for early clinical stage CRC. *SEPT9* as a DNA methylation biomarker was also associated with breast cancer; Gonzalez *et al* (32) indicated that increased *SEPT9* expression may contribute to the pathogenesis of certain types of breast cancer. Another potential biomarker for breast cancer is the methylation of *PITX2*. The evaluation of the *PITX2* methylation status among different breast cancer patient populations successfully increased the outcome prediction performance. Harbeck *et al* (33) investigated *PITX2* methylation in 399 breast cancer specimens and identified low-risk patients with hormone receptor-positive and node-negative disease. Hartmann *et al* (34) also analyzed the DNA methylation levels of *PITX2* in 241 breast cancer specimens and concluded that methylation of *PITX2* was correlated with clinical outcome. Hrasovec *et al* (35) reported that the alterations of CpG sites in *TMEM25* were correlated with CRC, with *TMEM25* hypermethylation possibly playing a significant role in altering the expression of this gene in CRC.

## 6. Conclusion

The elucidation of the mechanisms that underlie DNA methylation changes in cancer cells may help identify a number of cancer-specific methylation markers, assisted by promising detection methods, ultimately resulting in optimized clinical applications. The realization that DNA methylation may be involved in human malignancies and is ubiquitous in human diseases is likely to promote the development of novel diagnostic, preventive and therapeutic strategies.

The association between methylation and heredity is currently an emerging research topic. The presence of modified m5C affecting the phenotype of the offspring may be parentally inherited. Carone *et al* (36) indicated that parental diet may affect cholesterol and lipid metabolism in the offspring, defined a model system to study the environmental reprogramming of the heritable epigenome and concluded that epigenetic information may be inherited and represents environmental information. In another aspect, epigenetic information is one of the main areas of interest for the development of non-invasive prenatal diagnosis. Papageorgiou *et al* (37) reported the presence of epigenetic differences between placental and peripheral blood and identified a large number of previously unreported fetal epigenetic molecular markers that have the potential of being developed into targets for non-invasive prenatal diagnosis. Zhao *et al* (38) considered hypermethylated *RASSF1A* to be an epigenetic marker for the detection of fetal DNA in maternal plasma. Epigenetic information passed from parent to offspring and DNA methylation from gametes was shown to be predominantly maternal (39). Similar mechanisms maintaining the imprinted and non-imprinted methylation passed on from the mother represent an interesting focus of investigation and may be clinically applied for non-invasive prenatal diagnosis. Wang *et al* (40) recently reported that the hypermethylation status of the testis derived transcript gene promoter may represent a valuable prognostic marker for glioblastoma.

**Figure 1:**
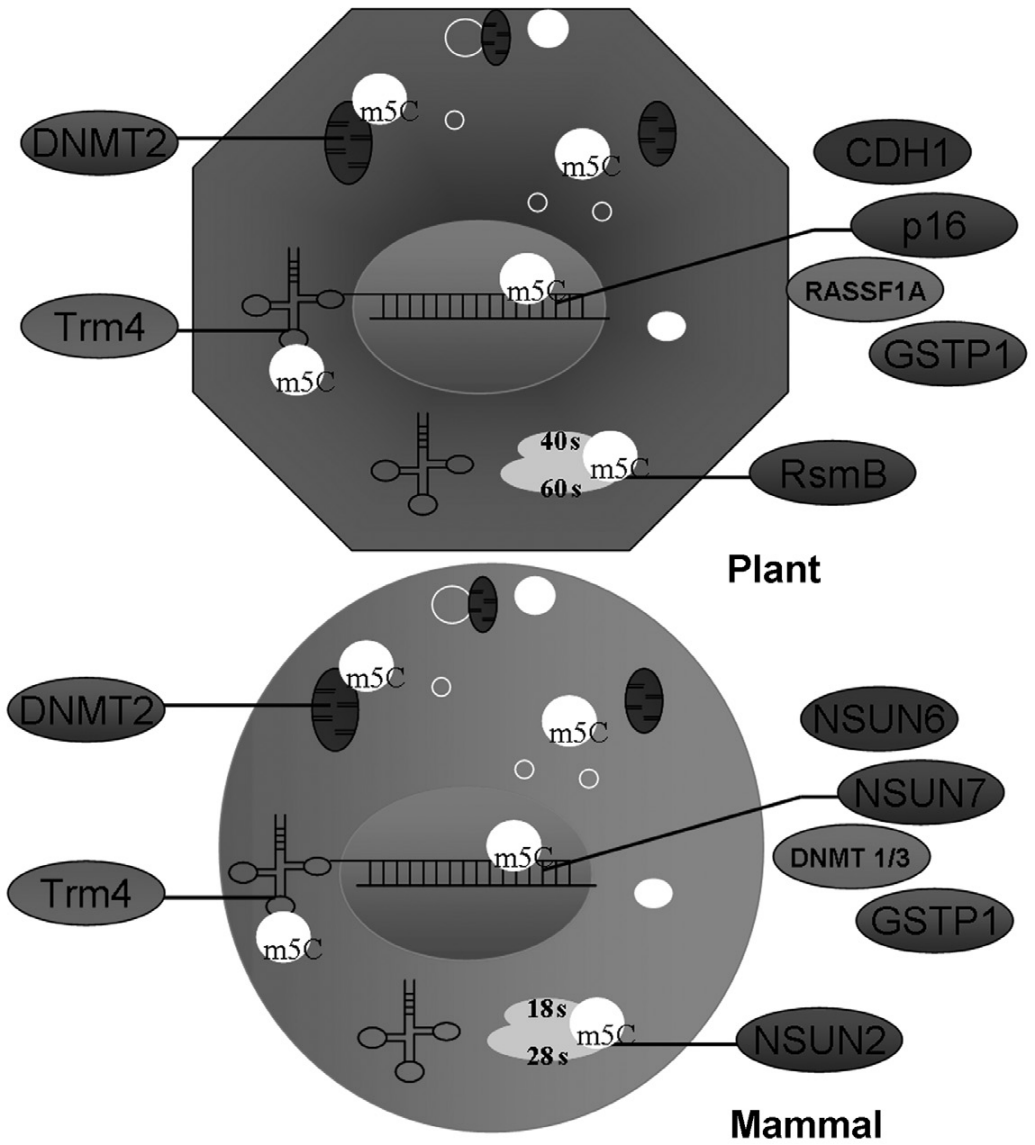
5-Methylcytosine (m5C) DNA and RNA residues in eukaryotes.

The modified DNA m5C that extensively occurs within the genome was extensively investigated over the last few decades; however, numerous putative methylated RNAs have been identified and characterized, regarding location, mechanism of formation and cellular function (41). Additional characterization of specific RNA methyltransferase enzymes and the association between DNA and RNA methylation is yet to be investigated (42). In eukaryotes, the majority of m5C methyltransferases are predicted to be nuclear or nucleolar proteins, which corresponds well to their functions in tRNA and rRNA processing (Fig. 1) (43). The expression of the human Trm4 (hTrm4) cDNA in yeast was identified, the first human gene encoding tRNA methylase responsible for the methylation of hTrm4 (44). Various regulatory RNAs, coding RNAs and newly discovered RNAs obtained based on high-throughput sequencing techniques may help elucidate the mechanisms of epigenetics (45).

## References

[1] Heyn H, Mendez-Gonzalez J, Esteller M (2013). Epigenetic profiling joins personalized cancer medicine. Expert Rev Mol Diagn.

[2] Smith RA, Brooks D, Cokkinides V, Saslow D, Brawley OW (2013). Cancer screening in the United States, 2013: a review of current American Cancer Society guidelines, current issues in cancer screening, and new guidance on cervical cancer screening and lung cancer screening. CA Cancer J Clin.

[3] Hanahan D, Weinberg RA (2011). Hallmarks of cancer: the next generation. Cell.

[4] Dawson MA, Kouzarides T (2012). Cancer epigenetics: from mechanism to therapy. Cell.

[5] Aran D, Hellman A (2013). DNA methylation of transcriptional enhancers and cancer predisposition. Cell.

[6] Esteller M (2007). Cancer epigenomics: DNA methylomes and histone-modification maps. Nat Rev Genet.

[7] Tolstorukov MY, Sansam CG, Lu P (2013). Swi/Snf chromatin remodeling/tumor suppressor complex establishes nucleosome occupancy at target promoters. Proc Natl Acad Sci USA.

[8] Pereira MA, Kramer PM, Conran PB (2001). Effect of chloroform on dichloroacetic acid and trichloroacetic acid-induced hypomethylation and expression of the c-myc gene and on their promotion of liver and kidney tumors in mice. Carcinogenesis.

[9] Fraga MF, Ballestar E, Paz MF (2005). Epigenetic differences arise during the lifetime of monozygotic twins. Proc Natl Acad Sci USA.

[10] Reik W, Collick A, Norris ML (1987). Genomic imprinting determines methylation of parental alleles in transgenic mice. Nature.

[11] Onyango P, Jiang S, Uejima H (2002). Monoallelic expression and methylation of imprinted genes in human and mouse embryonic germ cell lineages. Proc Natl Acad Sci USA.

[12] Tost J, Dunker J, Gut IG (2003). Analysis and quantification of multiple methylation variable positions in CpG islands by pyrosequencing. Biotechniques.

[13] Craig JM, Bickmore WA (1994). The distribution of CpG islands in mammalian chromosomes. Nat Genet.

[14] Gutierrez JC, Callejas S, Borniquel S (2000). DNA methylation in ciliates: implications in differentiation processes. Int Microbiol.

[15] Guintivano J, Arad M, Gould TD (2013). Antenatal prediction of postpartum depression with blood DNA methylation biomarkers. Mol Psychiatry.

[16] Di Ruscio A, Ebralidze AK, Benoukraf T (2013). DNMT1-interacting RNAs block gene-specific DNA methylation. Nature.

[17] Xie W, Schultz MD, Lister R (2013). Epigenomic analysis of multilineage differentiation of human embryonic stem cells. Cell.

[18] Chang X, Monitto CL, Demokan S (2010). Identification of hypermethylated genes associated with cisplatin resistance in human cancers. Cancer Res.

[19] Wu X, Rauch TA, Zhong X (2010). CpG island hypermethylation in human astrocytomas. Cancer Res.

[20] Daskalos A, Nikolaidis G, Xinarianos G, Savvari P, Cassidy A, Zakopoulou R, Kotsinas A, Gorgoulis V, Field JK, Liloglou T (2009). Hypomethylation of retrotransposable elements correlates with genomic instability in non-small cell lung cancer. Int J Cancer.

[21] Tan K, Kajino K, Momose S (2010). Mesothelin (MSLN) promoter is hypomethylated in malignant mesothelioma, but its expression is not associated with methylation status of the promoter. Hum Pathol.

[22] Hama R, Watanabe Y, Shinada K (2012). Characterization of DNA hypermethylation in two cases of peritoneal mesothelioma. Tumour Biol.

[23] Stenvinkel P, Karimi M, Johansson S (2007). Impact of inflammation on epigenetic DNA methylation - a novel risk factor for cardiovascular disease?. J Intern Med.

[24] Obeid R, Schadt A, Dillmann U (2009). Methylation status and neurodegenerative markers in Parkinson disease. Clin Chem.

[25] Laird PW (2010). Principles and challenges of genome-wide DNA methylation analysis. Nat Rev Genet.

[26] Riggs AD, Jones PA (1983). 5-methylcytosine, gene regulation, and cancer. Adv Cancer Res.

[27] Jin Z, Cheng Y, Gu W (2009). A multicenter, double-blinded validation study of methylation biomarkers for progression prediction in Barrett’s esophagus. Cancer Res.

[28] Martinez-Galan J, Torres B, Del Moral R (2008). Quantitative detection of methylated ESR1 and 14-3-3-sigma gene promoters in serum as candidate biomarkers for diagnosis of breast cancer and evaluation of treatment efficacy. Cancer Biol Ther.

[29] Glockner SC, Dhir M, Yi JM (2009). Methylation of TFPI2 in stool DNA: a potential novel biomarker for the detection of colorectal cancer. Cancer Res.

[30] Choi JY, James SR, Link PA (2009). Association between global DNA hypomethylation in leukocytes and risk of breast cancer. Carcinogenesis.

[31] Chen WD, Han ZJ, Skoletsky J (2005). Detection in fecal DNA of colon cancer-specific methylation of the nonexpressed vimentin gene. J Natl Cancer Inst.

[32] Gonzalez ME, Peterson EA, Privette LM (2007). High SEPT9_v1 expression in human breast cancer cells is associated with oncogenic phenotypes. Cancer Res.

[33] Harbeck N, Nimmrich I, Hartmann A (2008). Multicenter study using paraffin-embedded tumor tissue testing PITX2 DNA methylation as a marker for outcome prediction in tamoxifen-treated, node-negative breast cancer patients. J Clin Oncol.

[34] Hartmann O, Spyratos F, Harbeck N (2009). DNA methylation markers predict outcome in node-positive, estrogen receptor-positive breast cancer with adjuvant anthracycline-based chemotherapy. Clin Cancer Res.

[35] Hrasovec S, Hauptman N, Glavac D (2013). TMEM25 is a candidate biomarker methylated and down-regulated in colorectal cancer. Dis Markers.

[36] Carone BR, Fauquier L, Habib N (2010). Paternally induced transgenerational environmental reprogramming of metabolic gene expression in mammals. Cell.

[37] Papageorgiou EA, Fiegler H, Rakyan V (2009). Sites of differential DNA methylation between placenta and peripheral blood: molecular markers for noninvasive prenatal diagnosis of aneuploidies. Am J Pathol.

[38] Zhao F, Wang J, Liu R (2010). Quantification and application of the placental epigenetic signature of the RASSF1A gene in maternal plasma. Prenat Diagn.

[39] Muers M (2011). Methylation from mother. Nat Rev Genet.

[40] Wang LJ, Bai Y, Bao ZS (2013). Hypermethylation of testis derived transcript gene promoter significantly correlates with worse outcomes in glioblastoma patients. Chin Med J (Engl).

[41] Zheng G, Dahl JA, Niu Y (2013). ALKBH5 is a mammalian RNA demethylase that impacts RNA metabolism and mouse fertility. Mol Cell.

[42] Motorin Y, Lyko F, Helm M (2010). 5-methylcytosine in RNA: detection, enzymatic formation and biological functions. Nucleic Acids Res.

[43] Jurkowski TP, Jeltsch A (2011). On the evolutionary origin of eukaryotic DNA methyltransferases and Dnmt2. PLoS One.

[44] Brzezicha B, Schmidt M, Makalowska I (2006). Identification of human tRNA:m5C methyltransferase catalysing intron-dependent m5C formation in the first position of the anticodon of the pre-tRNA Leu (CAA). Nucleic Acids Res.

[45] Stevens M, Cheng JB, Li D (2013). Estimating absolute methylation levels at single-CpG resolution from methylation enrichment and restriction enzyme sequencing methods. Genome Res.

